# The pro-tumoral function of HACE1

**DOI:** 10.18632/oncoscience.418

**Published:** 2018-06-23

**Authors:** Najla El-Hachem, Robert Ballotti

**Affiliations:** Team 1, Biology and pathologies of melanocytes, Equipe labellisée ARC 2015, Université Côte d'Azur, Inserm U1065, C3M, Nice, France

**Keywords:** ubiquitination, melanoma, fibronectin

HACE1 (HECT Domain and Ankyrin Repeat Containing E3 Ubiquitin Protein Ligase 1) is an E3 ubiquitin ligase composed of six N-terminal ankyrin repeats linked to a C-terminal HECT domain [1]. Ankyrin repeats allows substrate recognition, whereas the HECT domain is essential for ubiquitinylation. HACE1 is ubiquitously expressed in all normal human tissues, with elevated expression in the heart, brain, kidney and pancreas. It is mainly located in the endoplasmic reticulum, the Golgi [2] and the cytoplasm. Initially, the analysis of chromosome translocation in Wilms' tumors identified HACE1 as a tumor suppressor [1], and similar observations were made in other neoplasms, such as breast cancer, hepatocellular carcinoma, B and NK cell lymphomas. A HACE1 knock-out mouse model [3] also supported a tumor suppressor role for HACE1, however, tumors mainly developed in animals that had lost p53, another tumor suppressor. HACE1 expression is frequently downregulated in cancers due to chromosomic rearrangements or DNA methylation at the HACE1 locus, which offers further evidence for its tumor suppressor activity. Furthermore, HACE1 was found to be mutated in cancer tissues and these mutations appeared to dampen its tumor suppressor activity [4]. Parallel studies focusing on the mechanism of RAC1 degradation showed that HACE1 promoted poly-ubiquitination of GTP-RAC1 in K48, allowing its proteasomal degradation. Thus, HACE1 is a key player in the control of active RAC1 levels [5]. Given the extensive and compelling data demonstrating the pro-oncogenic function of RAC1, the role of HACE1 in the degradation of RAC1 is consistent with its tumor suppressor activity.

In melanoma, the RAC1 P29S mutation is one of the most common somatic mutations after BRAF^V600^ and NRAS^Q61^. In contrast to the BRAF or NRAS mutations, the *RAC1* mutation displays a signature of ultraviolet B radiation (C→T substitutions at dipyrimidine sites), which is the main etiological factor for melanoma. The P29S mutation is an oncogenic mutation that maintains RAC1 in a GTP-bound form and increases melanocyte proliferation and migration [6].

Therefore, we reasoned that HACE1, which controls RAC1 stability, would have a predominant tumor suppressor role in melanoma. Nevertheless, initial analysis of publicly available datasets showed that HACE1 expression was maintained in melanoma, at levels comparable to normal melanocytes or nevi, and HACE1 expression was even higher in patients with poor survival (GSE19234). Further, using multiple melanoma cell lines or short-term melanoma cell cultures, we demonstrated that HACE1 silencing inhibited melanoma cell adhesion and migration *in vitro*. Similarly, lung colonization was dramatically impaired in HACE1-deficient melanoma cells, while HACE1 over-expression increased the capacity of the poorly metastatic 501MEL cells to colonize the lungs. Taken together, these observations support a pro-tumorigenic role of HACE1 in melanoma. This unexpected result prompted us to investigate the molecular mechanisms that could explain the role of HACE1 in melanoma.

Transcriptomic analyses demonstrated that HACE1 silencing induced a modification in the gene expression profile characterized by the inhibition of a set of genes involved in cell movement, migration and invasion, including *ITGAV*, *ITGB1*, *PLAU* and *SDC4*. These genes are regulated by fibronectin but we did not observe any down-regulation of FN1 upon HACE1 silencing. However, we demonstrated that HACE1 inhibition decreased fibronectin secretion and identified fibronectin as a new HACE1 substrate. Interestingly, HACE1 does not promote the degradative K48 ubiquitination of fibronectin, rather, it induces K27 ubiquitination that controls fibronectin secretion. Similarly, HACE1 has been reported to control YB1 secretion through K27 ubiquitination [7].

Fibronectin needs to be secreted before it can interact with its receptors (integrins), and consequently activate downstream signalling pathways and regulate specific transcriptomic programs, including *ITGAV* and *ITGB1* expression. Therefore, by controlling fibronectin secretion, HACE1 is a key player in the regulation of a feed-forward loop between integrins and fibronectin that plays a key role in adhesion, migration and metastatic ability of melanoma cells.

Our data do not agree with the existing dogma that attributes a universal tumor suppressor and point out the existence of specific functions of HACE1 according to the cellular context. This hypothesis is strengthened by analysis of TCGA data sets, which show that HACE1 behaves as a tumor suppressor (individuals with the highest 20% of HACE1 expression have increased survival) in acute myeloid leukemia and in head and neck squamous carcinoma, while it has a pro-oncogenic function (individuals with the highest 20% of HACE1 expression have decreased survival) in sarcoma and uterine corpus endometrial carcinoma.

These apparent contradictory roles of HACE1 can be understood in light of its complex molecular functions. Indeed, HACE1 does not only target RAC1 to the proteasome, HACE1 also interacts with RAB 1, 4 and 11 GTPases and is involved in Golgi membrane fusion during the cell cycle [2], suggesting that HACE1 loss would impair Golgi functioning and cell division. The HACE1 protein has also been shown to promote K48 ubiquitination of optineurin for its targeting to the autophagic degradation [8]. However, in human lung cancer cells the tumor suppressor function of HACE1 appears to be very dependent on optineurin expression. It can be hypothesized that the interaction between HACE1 and optineurin is regulated by post-translational modifications, such as phosphorylation, which would consequently modulate the cellular functions of HACE1 according to the activation of specific signaling pathways. Finally, HACE1 also promotes the K63 ubiquitinylation of the TRAF2 adapter protein that is required for TNF-induced NFKB activation and apoptosis [9]. Again, HACE1 exhibits contradictory functions, as NFKB pathway is a known anti-apoptotic pathway.

To summarize, HACE1 is an E3 ligase that is involved in the activation of antagonistic cellular functions, and which drives K27, K48 and K63 ubiquitination on different substrates. Therefore, the role of HACE1 cannot be reduced merely to its effect on RAC1 degradation and the tumor suppressor function that has been observed in several neoplasms. Our work demonstrates a pro-tumoral role of HACE1 in melanoma cells and opens new avenues of research to decode the cell- or context-specific molecular cascades that orientate HACE1 towards a pro- or anti-tumoral role.
